# Predictive Factors of Surgical Site Infection Following Cranioplasty: A Study Including 3D Printed Implants

**DOI:** 10.3389/fneur.2021.745575

**Published:** 2021-11-02

**Authors:** Myung Ji Kim, Hae-Bin Lee, Sung-Kon Ha, Dong-Jun Lim, Sang-Dae Kim

**Affiliations:** Department of Neurosurgery, Korea University Medical Center, Korea University College of Medicine, Ansan Hospital, Ansan-si, South Korea

**Keywords:** autologous bone flap, complication, cranioplasty, polyetheretherketone (PEEK), polymethyl methacrylate (PMMA), surgical site infection, titanium

## Abstract

In patients who have undergone decompressive craniectomy (DC), subsequent cranioplasty is required to reconstruct cranial defects. Surgical site infection (SSI) following cranioplasty is a devastating complication that can lead to cranioplasty failure. The aim of the present study, therefore, was to identify predictive factors for SSI following cranioplasty by reviewing procedures performed over a 10-year period. A retrospective analysis was performed for all patients who underwent cranioplasty following DC between 2010 and 2020 at a single institution. The patients were divided into two groups, non-SSI and SSI, in order to identify clinical variables that are significantly correlated with SSI following cranioplasty. Cox proportional hazards regression analyses were then performed to identify predictive factors associated with SSI following cranioplasty. A total of 172 patients who underwent cranioplasty, including 48 who received customized three-dimensional (3D) printed implants, were enrolled in the present study. SSI occurred in 17 patients (9.9%). Statistically significant differences were detected between the non-SSI and SSI groups with respect to presence of fluid collections on CT scans before and after cranioplasty. Presence of fluid collections on computed tomography (CT) scan before (*p* = 0.0114) and after cranioplasty (*p* < 0.0000) showed significant association with event-free survival rate for SSI. In a univariate analysis, significant predictors for SSI were fluid collection before (*p* = 0.0172) and after (*p* < 0.0001) cranioplasty. In a multivariate analysis, only the presence of fluid collection after cranioplasty was significantly associated with the occurrence of SSI (*p* < 0.0001). The present study investigated predictive factors that may help identify patients at risk of SSI following cranioplasty and provide guidelines associated with the procedure. Based on the results of the present study, only the presence of fluid collection on CT scan after cranioplasty was significantly associated with the occurrence of SSI. Further investigation with long-term follow-up and large-scale prospective studies are needed to confirm our conclusions.

## Introduction

Cranioplasty is required to reconstruct cranial defects for patients undergoing decompressive craniectomy (DC) to treat refractory intracranial hypertension due to traumatic brain injury, cerebral infarction, intracranial hemorrhage, and various causes of brain edema, or craniectomy for compound comminuted depressed and/or open/contaminated skull fractures (1–5). Cranioplasty offers not only cerebral protection and cosmetic repair, but also restores the intracranial and atmospheric pressure balance, improving the flow dynamics of cerebrospinal fluid (CSF) (6–13). Although there is no clear consensus on which material is the most appropriate, several materials are used for cranioplasty, including autologous bone, polymethyl methacrylate (PMMA), hydroxyapatite cement, polyetheretherketone (PEEK), and titanium (8, 14–17). Recent advances in three-dimensional (3D) printing technology and medical imaging have enabled the production of custom-made prefabricated patient-specific synthetic implants, offering a precise fit (8, 18–21). Despite these advances and the technical simplicity of the procedure, cranioplasty is associated with a high rate of complications, such as infection, bone resorption, postoperative hemorrhage, seizure, and hydrocephalus, which can increase the morbidity and mortality (6, 8, 9, 14, 22). Surgical site infection (SSI) is a devastating complication ranges from 12.3 to 29.7% (1, 6, 22–24) that can lead to cranioplasty failure, additional surgery, and neurologic deterioration. The identification of possible predictive factors of SSI may help surgeons decide which materials to use, recognize at-risk patients, and guide prophylactic care. The aim of the present study was to identify predictive factors of SSI following cranioplasty by reviewing procedures performed over a 10-year period, including 172 cases, 48 of which involved the use of 3D printed patient-specific implants.

## Materials and Methods

### Patients

**Figure 1:**
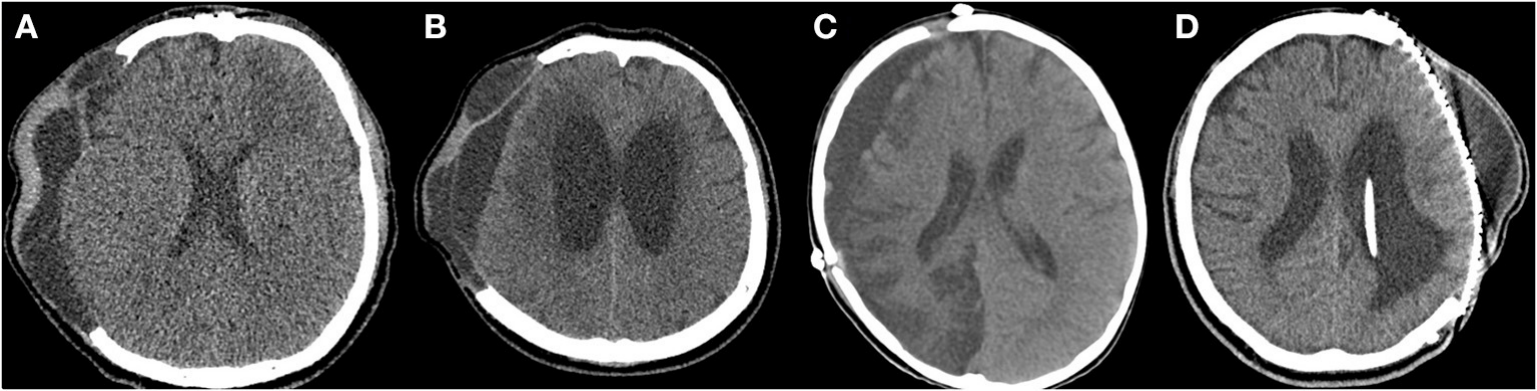
Preoperative and postoperative fluid collections on computed tomography (CT) scans. **(A)** Subgaleal and epidural fluid collections following decompressive craniectomy on the preoperative CT scan. **(B)** Subdural fluid collections with hydrocephalus on the preoperative CT scan. **(C)** Subdural fluid collection on the postoperative CT scan (7 days after cranioplasty). **(D)** Subgaleal fluid collection on the postoperative CT scan (7 days after cranioplasty).

A retrospective analysis was performed on all patients who underwent cranioplasty between 2010 and 2020 at a single institution, either following DC for traumatic brain hemorrhage or stroke, or craniectomy for brain tumor or compound comminuted depressed skull fractures. Clinical follow-up included a neurologic examination, evaluation of the wound, and radiologic assessment. All patients underwent pre- (1–2 days before cranioplasty) and postoperative (immediately after and 7 days after cranioplasty) computed tomography (CT) scans. Patients were excluded if the craniectomy was performed for infection, such as abscess, empyema, or postoperative infection, and patients with a follow-up period of <6 months after cranioplasty were also excluded. The following data were collected: demographics (age and sex), blood test, urine analysis, chest X-ray before cranioplasty, indication for initial craniectomy, time between craniectomy and cranioplasty, number of previous cranial surgeries, operative time, type(s) of material used for cranioplasty, SSIs, ventriculoperitoneal shunt (VPS) placement, medical comorbidity (hypertension [HTN], diabetes mellitus [DM], body mass index [BMI], and current smoking), pre- and postoperative Glasgow Outcome Scale (GOS) score (1 = dead, 2 = vegetative state, 3 = severe disability, 4 = moderate disability, and 5 = good recovery) (25), postoperative hematoma on CT scans, and pre- and/or postoperative fluid collections (subgaleal, epidural, and/or subdural) on CT scans. Fluid collections included subgaleal/epidural CSF, exudate from subgalea/muscle, or subdural hygroma (Figure 1). This study received ethical approval from the institutional review board of our institution (IRB number: 2021AS0136).

### Cranioplasty

**Figure 2:**
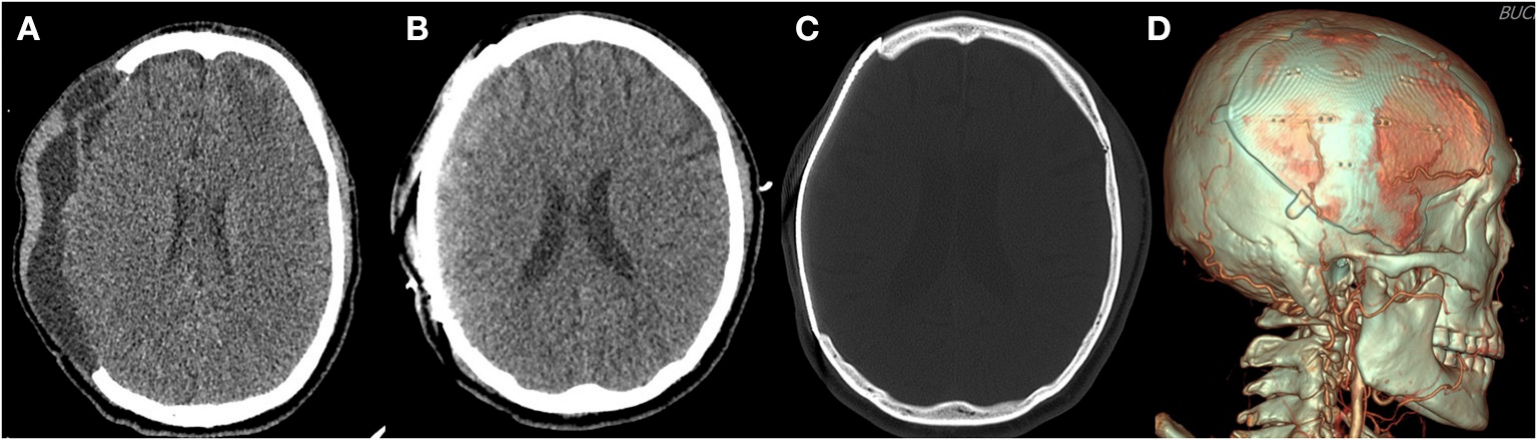
Three-dimensional (3D) printed patient-specific titanium implant. **(A)** The preoperative computed tomography scan of a patient with subgaleal and epidural fluid collection before cranioplasty. **(B)** The immediate postoperative computed tomography scan reveals no postoperative fluid collection after surgical evacuation during cranioplasty. **(C)** Bone setting view. **(D)** 3D reconstructed view.

**Figure 3:**
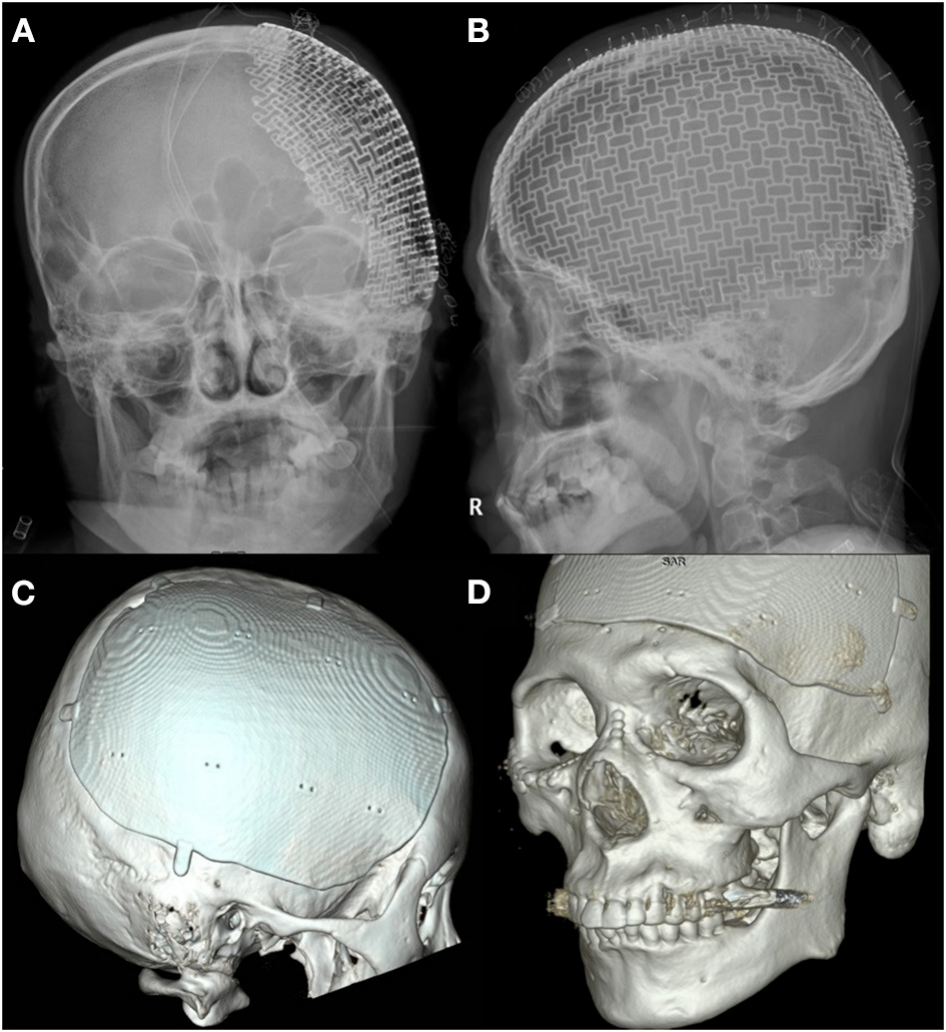
Advances in three-dimensional (3D) printed patient-specific titanium implant. **(A)** X-ray anteroposterior skull view of three-dimensional (3D) printed implant used in the beginning. **(B)** X-ray lateral skull view of 3D printed implant used in the beginning. **(C)** 3D reconstructed view of advanced 3D printed implant for the right frontotemporoparietal skull defect. **(D)** 3D reconstructed view of advanced 3D printed implant for the frontotemporal skull defect crossing the midline.

The cranioplasty procedure has been described in depth previously (26). In brief, soft tissue attached to autologous bone flaps (ABFs) was removed during craniectomy, ABFs were irrigated with normal saline, packed inside two sterile bags, and stored in a freezer at −80°C. The ABFs were soaked in a betadine solution during the dissection of the scalp flap and temporalis muscle from the underlying dura, and then fixed to the skull defect with multiple plates and screws. A titanium mesh was manually shaped during surgery, or PMMA was used if ABF was not available. Subgaleal drainage was routinely performed in all patients. DC and cranioplasty have been performed by three neurosurgeons in our center for the study period and there were not significant differences due to the procedure performed by different equips, except for the graft materials. Since 2017, 3D printed patient-specific implants (titanium) have been used for cranioplasty (Figures 2B–D, 3). Patients receiving 3D printed implants underwent preoperative 3D imaging CT scans, from which the skull defect was used as a template to create customized 3D printed flaps (14). Patients were classified into four groups, based on the material used for cranioplasty: autologous bone, PMMA, titanium mesh, and 3D printed titanium implant. Patients with hydrocephalus also underwent concurrent VPS placement. Routinely prophylactic antibiotics (2 g of cefazolin) were administered before skin incision and re-administered every 8 h after the operation until postoperative 2 days. If a patient was confirmed that there was a hypersensitivity to cefazolin through after skin test, 1 g of vancomycin was administered every 12 h until postoperative 2 days. If a patients scheduled for cranioplasty had a fever or signs of infection (pneumonia, urinary tract infection, blood stream infection), the procedure was delayed until fever subsided or infection was treated.

### Surgical Site Infection

SSI was defined as follows: (1) deep SSI requiring removal of ABF or implant due to purulent fluid discharge with signs of infection and complicated fluid collection, empyema, or abscess on CT scans; and (2) wound dehiscence with flap exposure requiring surgical revision without removal of ABF or implant (1, 13, 27). Patients who underwent cranioplasty were divided into two groups, non-SSI and SSI, in order to identify the clinical variables significantly correlated with SSI following cranioplasty.

### Statistical Analysis

Descriptive statistics are presented as the mean and standard deviation for continuous variables, and as frequencies and percentages for categorical variables. The chi-square test, Fisher exact test, and Mann-Whitney *U*-test were used to evaluate differences between the non-SSI and SSI groups. Kaplan-Meier survival analysis was used to investigate the association between clinical variables and the occurrence of SSI, after stratification using the log-rank test. Univariate and multivariate analyses were performed using the Cox proportional hazards regression model to analyze predictive factors associated with SSI following cranioplasty. Hazard ratios (HRs) and 95% confidence intervals (CIs) were calculated. Statistical significance was indicated by a *p* < 0.05, and all analyses were performed using statistical software (SAS version 9.4; SAS Inc., Cary, NC, USA; and R package, version 3.6.0).

## Results

### Patient Demographics

A total of 172 patients (126 men and 46 women) who underwent cranioplasty, including 48 who received customized 3D printed implants, were enrolled in the present study. Only three patients (1.7%) received vancomycin as prophylactic antibiotics, and the rest were routinely administered cefazolin, as previously mentioned. The patient demographics and surgery-specific factors are presented in Table 1. The mean interval between craniotomy and cranioplasty was 150.8 ± 366.0 days and the mean follow-up after cranioplasty was 22.0 ± 24.6 months. For cranioplasty, the materials used were as follows: 61.6% used ABFs, 27.9% used 3D printed implants, 7.6% used titanium mesh, and 2.9% used PMMA. Additionally, 9 (5.2%) patients underwent VPS placement prior to cranioplasty, and 34 (19.8%) underwent concurrent VPS and cranioplasty. Only five out of 34 patients who underwent concurrent VPS and cranioplasty had external hydrocephalus. The mean values of blood test performed before cranioplasty were within the normal range. The urine analysis revealed white blood cells in 8 patients (4.7%) and subsegmental atelectasis and mild pulmonary edema was confirmed in 6 patients (3.5%) on chest X-rays.

**Table 1:** Comparative baseline characteristics, patients-, and surgery-specific factors of all patients in the non-SSI and SSI following cranioplasty cohorts.

	**Total (*n* = 172)**	**Non-SSI (*n* = 155)**	**SSI (*n* = 17)**	***P*-value**
Mean age ± SD (years) Median (Q1, Q3)	46.5 ± 17.3 47.5 (38.5, 59)	46.4 ± 17.2 47 (38, 59)	47.1 ± 18.6 57 (39, 60)	0.646
Sex, *n* (%)				0.7777
Male	126 (73.3)	114 (73.6)	12 (70.6)	
Female	46 (26.7)	41 (26.4)	5 (29.4)	
Hypertension, *n* (%)	43 (25.0)	38 (24.5)	5 (29.4)	1.000
Diabetes mellitus, *n* (%)	24 (14.0)	20 (13.0)	4 (23.5)	0.2636
Mean body mass index ± SD (Kg/m^2^) Median (Q1, Q3)	23.4 ± 2.7 21.6 (20.8, 23.5)	23.5 ± 3.1 22.3 (21.4, 25.8)	21.2 ± 1.6 21.27 (19.8, 22.3)	0.054
Current smoking, *n* (%)	72 (41.9)	68 (43.9)	4 (23.5)	0.37
Hemoglobin ± SD (g/dL) Median (Q1, Q3)	11.9 ± 1.8 11.7 (10.3, 13.5)	12.4 ± 2.3 13.5 (10, 14.1)	11.3 ± 1.0 11.3 (10.5, 12.4)	0.216
White blood cell count ± SD (μL) Median (Q1, Q3)	5860.5 ± 1918.9 5,945 (4,230, 7,140)	5,558 ± 1353.4 5,115 (4,250, 7,020)	6,163 ± 2395.6 6,410 (4,160, 7,862)	0.496
Platelet count ± SD (μL) Median (Q1, Q3)	198,500 ± 74,716 205,000 (150,000, 258,000)	187,400 ± 70090.7 202,500 (120,250, 229,000)	209,600 ± 81232.5 207,000 (152,000, 268,250)	0.521
Erythrocyte sedimentation rate ± SD (mm/h) Median (Q1, Q3)	17.1 ± 12.3 17 (3.5, 29)	18.1 ± 13 19.5 (4.3, 30.1)	16.1 ± 12.2 17 (3, 25.8)	0.727
C-reactive protein ± SD (mg/dL) Median (Q1, Q3)	0.9 ± 2.2 0.1 (0.04, 0.2)	1.2 ± 2.8 0.12 (0.04, 0.64)	0.6 ± 1.5 0.12 (0.04, 0.36)	0.6
Indication for craniectomy, *n* (%)				0.5902
Trauma	106 (61.6)	92 (59.4)	14 (82.3)	
Subarchnoid hemorrhage	24 (14.0)	23 (14.9)	1 (5.9)	
Intracerebral hemorrhage	12 (7.0)	12 (7.7)	0 (0)	
Infarction	27 (15.7)	25 (16.1)	2 (11.8)	
Tumor	3 (1.7)	3 (1.9)	0 (0)	
Mean interval between craniectomy and cranioplasty ± SD (days) Median (Q1, Q3)	150.8 ± 366.0 59 (41.5, 98)	155.7 ± 384.0 58 (41, 97)	106.2 ± 101.5 63 (52, 121)	0.4044
Mean follow-up after cranioplasty ± SD (months) Median (Q1, Q3)	22.0 ± 24.6 14 (4, 30.5)	22.8 ± 24.8 15 (5, 31)	14.7 ± 22.4 4.7 (2.3, 15.4)	**0.0259**
Graft material, *n* (%)				0.0546
Autologous bone flap	106 (61.6)	93 (60.0)	13 (76.5)	
PMMA	5 (2.9)	5 (3.2)	0 (0)	
Titanium mesh	13 (7.6)	10 (6.5)	3 (17.6)	
3D printed implant	48 (27.9)	47 (30.3)	1 (5.9)	
Mean duration of cranioplasty ± SD (minutes)	129.9 ± 78.1	130.1 ± 80.6	128.3 ± 51.1	0.5989
Number of previous operations before cranioplasty, *n* (%)				0.8084
1	142 (82.6)	128 (82.6)	14 (82.4)	
2	27 (15.7)	24 (15.5)	3 (17.6)	
3	3 (1.7)	3 (1.9)	0 (0)	
Ventriculoperitoneal shunt, *n* (%)				0.8988
Placed before cranioplasty	9 (5.2)	9 (5.8)	0 (0)	
Placed at time of cranioplasty	34 (19.8)	31 (20.0)	3 (17.7)	
No shunt	129 (75.0)	115 (74.2)	14 (82.3)	
GOS score at time of cranioplasty, *n* (%)				0.6502
2		30 (19.4)	3 (17.6)	
3		30 (19.4)	4 (23.5)	
4		57 (36.7)	8 (47.1)	
5		38 (24.5)	2 (11.8)	
GOS score after cranioplasty, *n* (%)				0.2574
2		30 (19.4)	3 (17.7)	
3		14 (9.0)	3 (17.7)	
4		44 (28.4)	7 (41.1)	
5		97 (43.2)	4 (23.5)	
Fluid collection on CT scan before cranioplasty, *n* (%)	57 (33.1)	46 (29.7)	11 (64.7)	**0.0036**
Fluid collection on CT scan after cranioplasty, *n* (%)	26 (15.1)	13 (8.4)	13 (76.5)	**<0.0001**
Hemorrhage in CT scan after cranioplasty, *n* (%)	25 (14.5)	24 (15.5)	1 (5.9)	0.4727

*CT, computed tomography; GOS, Glasgow Outcome Score; PMMA, polymethyl methacrylate; SD, standard deviation; SSI, surgical site infection*.

*Boldface type indicates statistical significance (p < 0.05)*.

### Surgical Site Infection

**Figure 4:**
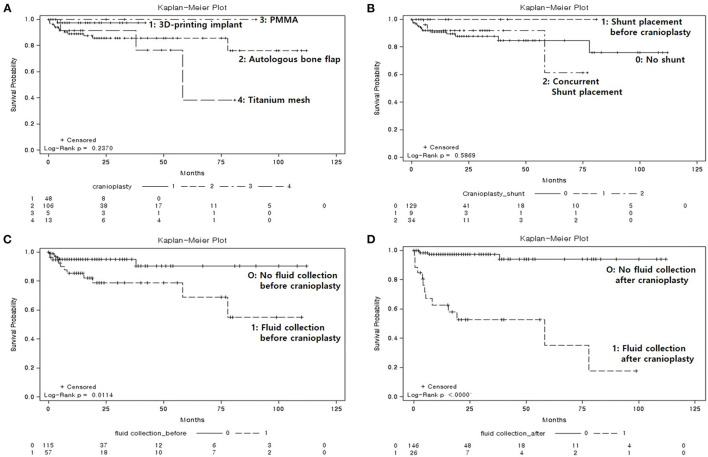
Kaplan-Meier event-free survival curves for post-cranioplasty surgical site infection, according to clinical variables. **(A)** Graft materials (*p* = 0.2370), 1: three-dimensional (3D) printed implant, 2: autologous bone flap, 3: polymethyl methacrylate, 4: titanium mesh. **(B)** Ventriculoperitoneal shunt (*p* = 0.5869), 0: no shunt, 1: shunt in place prior to cranioplasty, 2: concurrent shunt placement. **(C)** Fluid collection on computed tomography (CT) scan prior to cranioplasty (*p* = 0.0114), 0: no fluid collection, 1: fluid collection present. **(D)** Fluid collection on CT scan after cranioplasty (*p* < 0.0000), 0: no fluid collection, 1: fluid collection present.

SSIs occurred in 17 patients (9.9%), of which 13 (7.6%) required removal of the bone graft, and 4 (2.3%) required wound revision without removal of implant due to wound dehiscence with flap exposure. The time interval between cranioplasty and occurrence of SSI was 539 ± 773.3 days (range, 14–2,333 days). Patients with SSIs were compared with those who did not have SSIs (Table 1). Interestingly, the mean follow-up after cranioplasty was significantly longer in non-SSI group compared with SSI group mainly due to follow-up loss (non-SSI vs. SSI, 22.8 ± 24.8 vs. 14.7 ± 22.4 months, *p* = 0.00259). Statistically significant differences were found between the two groups with respect to the presence of fluid collections on CT scans before and after cranioplasty (non-SSI vs. SSI, 29.7 vs. 64.7%, *p* = 0.0036, and 8.4 vs. 76.5%, *p* < 0.0001, respectively). Among 57 patients whose fluid collections were observed on CT scans before cranioplasty, 37 patients had fluid collection removed spontaneously during the dissection of subgalea and muscle or by surgical evacuation, and only one of 37 patients developed SSI. On the other hand, there was no fluid collection before cranioplasty, but occurred after cranioplasty in a total of six patients, and three of them developed SSI. None of the patient- or surgery-specific characteristics differed significantly between the two groups. However, non-SSI group had a higher body mass index (BMI) at the time of surgery than the SSI group, although it was not statistically significant (non-SSI vs. SSI, 23.5 ± 3.1 vs. 21.2 ± 1.6, *p* = 0.054). Kaplan-Meier curves that evaluated the clinical variables in relation to SSI are displayed in Figure 4. There were no significant differences between graft materials (*p* = 0.2370; Figure 4A) and VPS (*p* = 0.5869; Figure 4B) for the event-free survival rate for SSI following cranioplasty. The presence of fluid collections on CT scans before (Figure 4C) and after (Figure 4D) cranioplasty showed a significant association with event-free survival rate for SSI (*p* = 0.0114 and *p* ≤ 0.0000, respectively). The log-rank test for sex (*p* = 0.9666), indication for craniectomy (*p* = 0.2941), DM (*p* = 0.4652), hemorrhage after cranioplasty (*p* = 0.4797), number of operations (*p* = 0.7804), GOS score before cranioplasty (*p* = 0.7940), and GOS score after cranioplasty (*p* = 0.4520) were not significantly different between the groups.

### Predictive Factors of Surgical Site Infection

The Cox regression model for factors related to time from cranioplasty to SSI is shown in Table 2. In a univariate analysis, significant predictors for SSI were fluid collection before (*p* = 0.0172) and after (*p* < 0.0001) cranioplasty, as seen on CT imaging. Patients with fluid collections on CT scans before and after cranioplasty showed a significant correlation for the occurrence of SSI at their last follow-up. In a multivariate analysis, only the presence of fluid collection on CT scans after cranioplasty was significantly associated with the occurrence of SSI (*p* < 0.0001). Patient-specific risk factors, such as age, sex, HTN, DM, BMI, smoking, GOS score, and blood test results before cranioplasty and surgery-specific risk factors (indication for craniectomy, mean interval between craniectomy and cranioplasty, graft materials, mean duration of cranioplasty, and VPS), were not significantly correlated with SSI.

**Table 2:** Predictive factors for time to surgical site infection following cranioplasty in 172 patients: Cox regression.

**Factors**	**Univariate analysis**			**Multivariate analysis**		
	**HR**	**CI**	***P*-value**	**HR**	**CI**	***P*-value**
Age	1.009	(0.981, 1.038)	0.5439			
Sex (female)	1.023	(0.358, 2.919)	0.9665			
Hypertension	0.583	(0.075, 4.562)	0.6084			
Diabetes mellitus	1.526	(0.487, 4.788)	0.4684			
Mean body mass index	0.616	(0.350, 1.084)	0.0932			
Current smoking	0.286	(0.045, 1.821)	0.1855			
Hemoglobin	0.703	(0.405, 1.222)	0.2111			
White blood cell count	1.000	(1.000, 1.001)	0.4757			
Platelet count	1.000	(1.000, 1.000)	0.5012			
Erythrocyte sedimentation rate	0.986	(0.916, 1.061)	0.7124			
C-reactive protein	0.882	(0.560, 1.389)	0.5883			
Indication for craniectomy (trauma)			0.7908			
Subarchnoid hemorrhage	0.285	(0.037, 2.182)	0.2267			
Intracerebral hemorrhage	0		0.9917			
Infarction	0.636	(0.144, 2.808)	0.5500			
Tumor	0		0.9954			
Mean interval between craniectomy and cranioplasty	0.999	(0.997, 1.002)	0.6399			
Graft materials			0.4011			
(Autologous bone flap)						
3D printed implant	0.218	(0.028, 1.686)	0.1445			
PMMA	0		0.9927			
Titanium mesh	1.598	(0.453, 5.629)	0.4658			
Mean duration of cranioplasty	0.996	(0.989, 1.003)	0.2701			
Number of previous operations before cranioplasty	0.698	(0.216, 2.26)	0.549			
Ventriculoperitoneal shunt			0.9759			
(no shunt)						
Placed before cranioplasty	0		0.9936			
Placed at the time of cranioplasty	0.868	(0.247, 3.052)	0.8254			
GOS score at time of cranioplasty	0.911	(0.571, 1.451)	0.6939			
GOS score after cranioplasty	0.855	(0.57, 1.283)	0.4499			
Fluid collection before cranioplasty	3.372	(1.241, 9.161)	**0.0172**	0.804	(0.253, 2.556)	0.7117
Fluid collection after cranioplasty	18.164	(5.913, 55.799)	**<0.0001**	20.423	(5.655, 73.754)	**<0.0001**
Hemorrhage after cranioplasty	0.49	(0.065, 3.705)	0.4891			

*CI, confidence interval; DM, diabetes mellitus; GOS, Glasgow Outcome Score; HR, Hazard ratio; PMMA, polymethyl methacrylate*.

*Boldface type indicates statistical significance (p < 0.05)*.

## Discussion

In the present study, we identified significant differences in pre- and postoperative fluid collections between the non-SSI and SSI groups (Table 1), and found that the presence of fluid collections on CT images demonstrated a significant correlation with the event-free survival rate for SSI (Figures 4C,D). Furthermore, univariate analysis revealed that fluid collections were a significant predictor of SSI (Table 2). In multivariate analysis, however, the presence of fluid collections on CT scans after cranioplasty was the only factor found to correlate with the occurrence of SSI (Table 2). Fluid collections post-DC have a variety of sources, including: subgaleal/epidural fluid resulting from CSF leakage due to loose approximation of the dura or closure with artificial dural implants (28), accumulation of exudate from the dissected subgaleal region and muscle (29), and subdural hygroma or external hydrocephalus due to altered CSF hydrodynamics (30). This may be related to dead space that forms when the swelling in the brain has receded after the resolution of the edema (7). Most fluid collections regress spontaneously over time, and previous studies have suggested that cranioplasty may actually improve cerebral blood flow and CSF hydrodynamics, resulting in the resolution of fluid collections due to external hydrocephalus (31–33). However, one previous study suggested that cranioplasty might increase the risk of hydrocephalus when performed <90 days after initial craniectomy (34). Based on the results of the present study, one way to prevent the development of SSIs following cranioplasty is to ensure that there are no fluid collections post-cranioplasty, even if there were fluid collections before the operation. If fluid collections are identified on CT images prior to the cranioplasty, the surgical evacuation of fluid collections during cranioplasty (Figure 2) or concurrent VPS placement in cases of external hydrocephalus may be considered. The patient who underwent sugical evacuation of fluid collection did not develop surgical site infection until his last follow-up (13 months) (Figure 2).

Hydrocephalus has been reported to occur in 10–40% of patients who have undergone DC (10, 35). Permanent methods of CSF diversion, such as VPSs, are required if hydrocephalus persists even after intracerebral pressure management with external ventricular drainage during the acute phase. However, there are varying results regarding when VPS placement should be performed, and there is no clear consensus on the risk of complications, particularly for the development of SSIs with staged or concurrent cranioplasty and VPS placement. Previous studies have reported that concurrent VPS placement and cranioplasty resulted in an increased rate of SSIs compared with staged operations (36, 37). Contrarily, other studies have concluded that the rate of SSIs did not differ significantly between concurrent and staged surgeries for VPS placement and cranioplasty (9, 13, 14). Postoperative hydrocephalus requiring VPS placement was observed in 25% of the cases in the present study. Of the 43 patients who underwent VPS placement, nine underwent placement prior to cranioplasty, and 34 underwent concurrent cranioplasty and VPS placement. We evaluated the event-free survival among patients who had undergone VPS placement prior to cranioplasty, concurrent VPS placement and cranioplasty, and no VPS placement. We identified no significant differences in the event-free survival rates for SSIs among these groups (Figure 4B). From the standpoint that concurrent VPS placement and cranioplasty increases the occurrence of SSIs, the cause of this is thought to be the negative gradient force induced by the over drainage of CSF through the VPS (21, 38). The negative gradient force causes not only a more depressed brain with postoperative dead space, but also pulls at the skin, leading to the exposure of graft materials (39). In the present study only five out of 34 patients who underwent concurrent VPS and cranioplasty had external hydrocephalus. Although the case was too small to analyze the statistical significance separately, four out of five patients improved external hydrocephalus and did not develop SSI during the follow-up period, but one patient did not improve external hydrocephalus and developed SSI. Although VPS placement was not a statistically significant variable that increased or decreased the risk of SSI in the present study, we still suggest that patients with persistent hydrocephalus, especially those with external hydrocephalus, undergo concurrent VPS placement, and cranioplasty. However, we also suggest that the pressure should be slowly adjusted via a programmable valve, to prevent excessive drainage of the CSF.

The present study evaluated the risk of SSI following cranioplasty among different graft materials, including 48 custom-made 3D printed implants. Traditionally, the material of choice for cranioplasty has been the patient's own preserved bone flap, due to a decreased risk of excessive immune response to foreign materials, and its ability to undergo bony regrowth and revascularization (18, 40). However, previous studies have demonstrated that ABF is correlated with the development of SSIs as well as bone flap resorption, resulting in graft failure (41, 42). This may be due to the denaturation of ABF, depending on the storage method (24). There are still no standard guidelines for the sterilization and preservation of ABFs. The largest study concerning predictors of infection after craniolasty by Morton et al. assessed the predictive value of intraoperative bone flap cultures, which are not performed in our center. The authors suggested that intraoperative bone cultures in the absence of infection should be discontinued since the culture results were not a reliable predictor of postcranioplasty infection in their analysis (43). The most commonly used techniques to preserve ABFs are cryopreservation and subcutaneous implantation of the flap into an abdominal pocket (44, 45). Our center has traditionally performed cryopreservation, via direct freezing at −80°C; however, recently, ABFs have not been as widely used for cranioplasty, and 3D printed implants have been used for 27.9% of the total number of cranioplasties performed at our center. In the present study, the most commonly used materials for cranioplasty were ABF (61.6%), 3D printed implants (titanium, *n* = 48) (27.9%), titanium mesh (7.6%), and PMMA (2.9%), was and we found no significant differences between the graft materials for the event-free survival rates for SSIs following cranioplasty (Figure 4A). Additionally, graft materials were not found to correlate with the occurrence of SSI following cranioplasty (Table 2). This finding was consistent with that of meta-analyses conducted by Yadla et al. (40) and Punchak et al. (46). Various synthetic materials have been used for cranioplasty, including PMMA, ceramics, hydroxyapatite, PEEK, and titanium. Each material has advantages and disadvantages, and as of yet there is no consensus on which one is most ideal for cranioplasty. Titanium provides a strong and non-corrosive material that can be manually shaped during operation; however, thermal conductivity, radiopacity, risk of metal hypersensitivity, and abrasiveness to overlying soft tissues are disadvantages of this material (8, 47). One study suggested that cranioplasty using titanium can greatly increase the implant exposure rate (2). Benefits of PEEK include chemical inertness, robustness, comfort, radiolucency, and thermal non-conductivity (20); however, extrusion has been reported due to the incorporation of bone defects. Synthetic materials are considered to have a higher risk of infection following cranioplasty, but the present study revealed that graft materials were not predictive factors for SSI, although we did demonstrate the superiority of 3D printed implants in providing a precise fit and satisfactory esthetic results. The 3D printed implant used in the early days required multiple screws to secure it to the bone (Figures 3A,B), but as 3D printing technology advanced, not only did the implant fit perfectly, but a screw fixing part and a tenting part to prevent epidural hemorrhage were created and provided (Figures 3C,D). In the present study, we identified the safety of patient-specific 3D printed implants; however, prefabricated implants are still expensive, and long-term complications remain to be investigated. Further investigations with continuous follow-up are necessary to confirm the long-term safety of 3D printed implants in the setting of cranioplasty.

### Limitations

The primary limitation of the present study is that it is a retrospective review of procedures performed at a single institution. Selection bias may have played a critical role in patient selection and the decision to perform surgery, because cranioplasty is performed based on the surgeons preferences, and only in patients who survive after DC, regardless of the indication for DC or neurologic deficits. Additionally, the treatment bias associated with the selection of graft materials based on “availability” was unavoidable. Further investigation with long-term follow-up and larger-scale studies is needed to confirm our conclusions.

## Conclusions

The present study investigated predictive factors that may help identify patients at risk of SSI following cranioplasty and provide guidelines associated with the procedure. The presence of fluid collections on CT scans before and after cranioplasty showed a significant association with the event-free survival rate for SSI. In the univariate analysis, the presence of fluid collections before and after cranioplasty was also a significant predictor for SSI. In the multivariate analysis, however, only the presence of fluid collections on CT scans after cranioplasty was significantly associated with the occurrence of SSIs. Surgery-specific risk factors, including graft materials and VPS placement, did not demonstrate a significant correlation with SSI. The surgical evacuation of fluid collections during cranioplasty or concurrent VPS placement may be considered in cases of external hydrocephalus as a way to reduce fluid collections present prior to cranioplasty. Further prospectively designed studies with long-term follow-term are needed to confirm our conclusions.

## Data Availability Statement

The raw data supporting the conclusions of this article will be made available by the authors, without undue reservation.

## Ethics Statement

The studies involving human participants were reviewed and approved by Korea University Ansan Hospital Institutional Review Board. Written informed consent for participation was not required for this study in accordance with the national legislation and the institutional requirements.

## Author Contributions

S-DK: conceptualization, writing—review and editing, and resources. MK: visualization and roles/writing—original draft. H-BL: data curation and formal analysis. S-KH: project administration investigation. D-JL: supervision and validation. All authors contributed to the article and approved the submitted version.

## Funding

This work was supported by Korea University (Grant Numbers K2101781 and K2118811) and Korea University Ansan Hospital (Grant Number O2105791).

## Conflict of Interest

The authors declare that the research was conducted in the absence of any commercial or financial relationships that could be construed as a potential conflict of interest.

## Publisher's Note

All claims expressed in this article are solely those of the authors and do not necessarily represent those of their affiliated organizations, or those of the publisher, the editors and the reviewers. Any product that may be evaluated in this article, or claim that may be made by its manufacturer, is not guaranteed or endorsed by the publisher.

## Abbreviations

ABF: Autologous bone flaps
BMI: Body mass index
CSF: cerebrovascular fluid
CT: Computed tomography
DC: Decompressive craniectomy
DM: Diabetes mellitus
GOS: Glasgow Outcome Scale
HTN: Hypertension
PEEK: Polyetheretherketone
PMMA: Polymethyl methacrylate
SSI: Surgical site infection
VPS: Ventriculoperitoneal shunt
3D: three-dimensional.
